# Swelling and Mechanical Characterization of Polyelectrolyte Hydrogels as Potential Synthetic Cartilage Substitute Materials

**DOI:** 10.3390/gels8050296

**Published:** 2022-05-12

**Authors:** Johanna Romischke, Anton Scherkus, Michael Saemann, Simone Krueger, Rainer Bader, Udo Kragl, Johanna Meyer

**Affiliations:** 1 Industrial Chemistry, Institute of Chemistry, University of Rostock, Albert-Einstein-Str. 3a, 18059 Rostock, Germany; johanna.romischke@uni-rostock.de (J.R.); anton.scherkus@uni-rostock.de (A.S.); udo.kragl@uni-rostock.de (U.K.); 2 Biomechanics and Implant Technology Research Laboratory, Department of Orthopaedics, Rostock University Medical Center, 18057 Rostock, Germany; michael.saemann@med.uni-rostock.de (M.S.); simone.krueger@uni-rostock.de (S.K.); rainer.bader@med.uni-rostock.de (R.B.); 3 Department Life, Light & Matter, Faculty for Interdisciplinary Research, University of Rostock, Albert-Einstein-Str. 25, 18059 Rostock, Germany; 4 Institute of Technical Chemistry, Leibniz University Hannover, Callinstraße 3-9, 30167 Hannover, Germany

**Keywords:** hydrogel, polyelectrolyte, synthetic polymer, swelling, mechanical characterization, compression, stress-relaxation, cyclic loading, cartilage lesion, substitute material

## Abstract

Hydrogels have become an increasingly interesting topic in numerous fields of application. In addition to their use as immobilization matrixes in (bio)catalysis, they are widely used in the medical sector, e.g., in drug delivery systems, contact lenses, biosensors, electrodes, and tissue engineering. Cartilage tissue engineering hydrogels from natural origins, such as collagen, hyaluronic acid, and gelatin, are widely known for their good biocompatibility. However, they often lack stability, reproducibility, and mechanical strength. Synthetic hydrogels, on the other hand, can have the advantage of tunable swelling and mechanical properties, as well as good reproducibility and lower costs. In this study, we investigated the swelling and mechanical properties of synthetic polyelectrolyte hydrogels. The resulting characteristics such as swelling degree, stiffness, stress, as well as stress-relaxation and cyclic loading behavior, were compared to a commercially available biomaterial, the ChondroFiller^®^ liquid, which is already used to treat articular cartilage lesions. Worth mentioning are the observed good reproducibility and high mechanical strength of the synthetic hydrogels. We managed to synthesize hydrogels with a wide range of compressive moduli from 2.5 ± 0.1 to 1708.7 ± 67.7 kPa, which addresses the span of human articular cartilage.

## 1. Introduction

Human cartilage is a connective tissue that can sustain high mechanical load and provides exceptional lubrication. Articular cartilage is composed mainly of chondrocytes located in a hydrated extracellular matrix (ECM) composed largely of type II collagen and proteoglycans [1]. The structure of cartilage is based on four layers that are distinguished by the orientation of collagen fibers and the composition of proteoglycans [2,3]. Due to trauma, arthritis, sports injuries, or natural degradation, cartilage can suffer from significant damage. The number of patients with cartilage lesions and clinical symptoms is increasing due to aging and obesity [4,5,6]. As a consequence of its avascular nature and low cell density, a self-healing process of cartilage occurs only to a limited extent, which is why a treatment method for permanent recovery is of considerable interest [6]. Damaged cartilage can be surgically repaired or replaced to relieve joint pain or restore joint function [7]. Clinical methods to repair cartilage lesions, including autologous chondrocyte implantation, micro fracturing, and mosaicplasty, have limited ability to regenerate functional cartilage due to a mismatch of composition and mechanical behavior of native and artificial cartilage [1,5,8].

Tissue engineering fills the gap by developing biomaterials that replace small areas of damaged tissue after implantation or injection or act as a three-dimensional network to carry active agents for regeneration [9]. The requirements for these scaffolds are multifaceted, such as adequate nutrient transport, proper cell adhesion, minimally invasive implantation procedure, low swelling behavior of <10%, and, if used as a regeneration support material, should degrade after 6 to 8 weeks at the earliest [1]. Most important, however, are the mechanical functions of the scaffolds. To describe the biomechanical properties of articular cartilage, the compressive, tensile and shear moduli, as well as Poisson’s ratio and permeability of the material, are usually determined [10]. However, these properties are subject to individual and species-specific variations, resulting in wide variations in literature values for biomechanical parameters of articular cartilage. In addition, cartilage also becomes more hardened when exposed to high pressure or when the joint fluid is reduced [10]. In tissue engineering, natural polymers, such as hyaluronic acid, chitosan, or alginate, are often used, due to their mostly good biocompatibility and minimal inflammatory or immunological reactions to the target tissue [9,11,12]. In general, the degradable polymers are mainly used as cartilage implants to deliver cells and growth factors and to stimulate or even accelerate the differentiation and proliferation of cartilage cells on or in the scaffold. However, these natural polymers usually have insufficient mechanical properties, which are, however, of enormous importance for the use of articular cartilage that is exposed to high loads. These biomaterials can be chemically modified to improve the bioactivity, strength, and elastic properties of the implants [5]. Most of these natural biopolymers possess functional groups such as amino, hydroxyl, carboxyl, sulfate and/or phosphate groups, and therefore most modifications are based on electrostatic interactions, hydrogen bonding, or esterification.

Compared to natural biomaterials, synthetic materials allow better reproducibility and controllability, as well as tunability, and often better mechanical properties. Mostly, polyethylene glycol (PEG)- or methacrylate-based compounds are used for this purpose, as they can be easily functionalized and have shown outstanding performance in the synthesis of hydrogels [1,6,13]. PEG-based hydrogels are relatively inert, biocompatible, and have been shown to support the formation of cartilage tissue. However, the entire structure of the gels must always be considered, because the choice of crosslinkers also influences the material properties. Roberts and Bryant et al. investigated PEG-based hydrogels for the development of cartilage substitute materials. The photopolymerized gels were crosslinked with acrylates on the one hand, but also with thiol-norbornene bridges on the other, resulting in improved mechanical properties and tissue similar to hyaline-like cartilage [14].

In another study, Bonakdar et al. successfully synthesized polyvinyl hydrogels that were crosslinked with biodegradable polyurethane. Both the mechanical and physical properties of this gel, suggest it as a possible candidate as cartilage replacement material. All samples had a compressive modulus in the range of cartilage (1.9–14.4 MPa), and by increasing the concentration of the hydrophobic polyurethane, the water absorption of the gels could be reduced to a cartilage-like ratio [15]. Yang et al. investigated the first synthetic hydrogel based on a poly(vinyl alcohol)(PVA)-poly(2-acrylamido-2-methyl-1-propanesulfonic acid sodium salt) (AAMPSO_3_), which exhibits the mechanical strength and Young’s modulus of human articular cartilage in both tension and compression [16].

Nevertheless, there is a great need for synthetic materials as substitute materials, which could eventually also act as combination materials for natural polymers. Especially in the case of polyelectrolyte hydrogels, crosslinked gels with charged repeating units at the polymer backbone exhibit outstanding intrinsic antibacterial properties, biocompatibility, or even the adhesion to glass, skin, or organs [17,18]. Wang et al. published a zwitterionic polyelectrolyte hydrogel and tuned the cell adhesion and antibacterial properties by introducing additional ionic monomers to the network [17]. In previous studies, we investigated the intrinsic antibacterial behavior of 11 different hydrogels as well as their corresponding monomers against *Staphylococcus aureus* Xen 30 (MRSA Xen 30) and *Pseudomonas aeruginosa* Xen 5 (*P. aeruginosa* Xen 5), including the polyelectrolyte hydrogels described in this work [19]. These hydrogels have cationic or anionic monomers as a repeating unit of the backbone, which is crosslinked to a 3D network. The hydrogels mentioned in this paper, such as 2-acrylamido-2-methyl-1-propanesulfonic acid (AAMPSO_3_) and [2-(methacryloyloxy)ethyl]trimethylammonium chloride (MATMA), were able to achieve up to 90%. These findings were very promising for the development of new antibacterial biomaterials for medical applications, for example, cartilage substitute materials.

In this work, these before-described antibacterial hydrogels based on polyelectrolytes, such as polyAAMPSO_3_ and polyMATMA, were synthesized and intensively characterized in their swelling behavior and mechanical properties. These materials were compared to the ChondroFiller^®^ liquid, a biomaterial already used for the treatment of cartilage lesions, showing promising performance, especially regarding mechanical properties. These findings could pave the way for more durable and easy-to-synthesize biomaterials as cartilage substitutes.

## 2. Results and Discussion

### 2.1. Gravimetric and Volumetric Swelling Experiments

**Figure 1:**
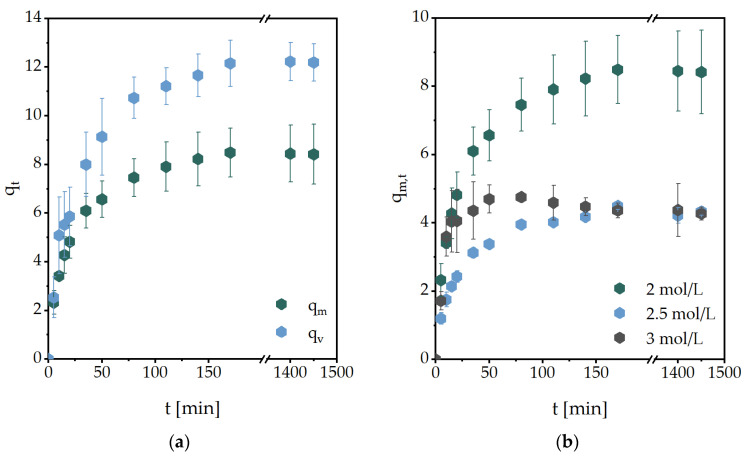

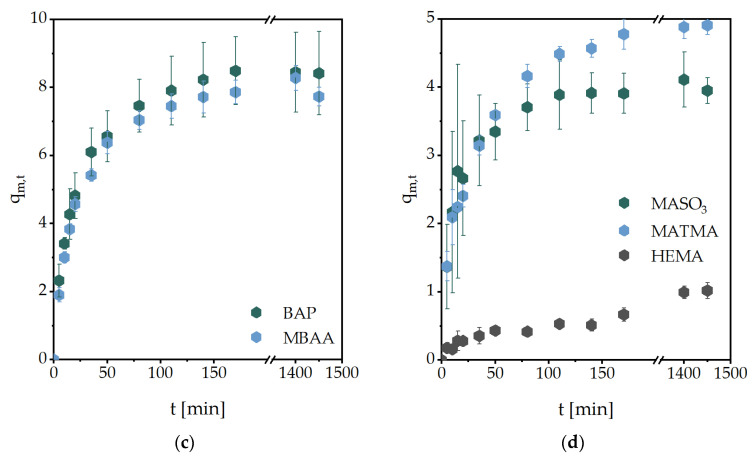
Water swelling kinetics of (**a**) MASO_3_ (2 mol/L) hydrogels with BAP (2 mol%) as a crosslinker, (**b**) MASO_3_ with different monomer concentrations and BAP as a crosslinker, (**c**) MASO_3_ (2 mol/L) and different crosslinkers (2 mol%) and (**d**) hydrogels with different monomer structures (3 mol/L) and BAP (2 mol%) as a crosslinker. All measurements were performed in triplicate, at 36 ± 1 °C in PBS.

One of the main properties of hydrogels is the ability to swell and expand in hydrophilic environments without losing their three-dimensional shape. In general, the swelling of a gel depends on the solvent mobility, solvent-polymer-interaction, polymer relaxation time, structure of the used monomer, the chain length of the crosslinker, and the degree of crosslinking [20]. In Figure 1, exemplary swelling kinetics of the investigated hydrogels are shown.

In Figure 1a, the volumetric (*q_v,t_*) and gravimetric (*q_m,t_*) swelling degree of MASO_3_ with BAP as a crosslinker is shown. It can be determined that the curves of the swellings are very similar, but the values of *q_v,t_* are continuously higher. Furthermore, an increase in the monomer concentration in the hydrogel leads to a decrease in the swelling degree (Figure 1b). However, if the monomer concentration is further increased, the swelling degree remains stable. Presumably, the hydrophilicity of the side chains continues to cause the hydrogel to swell. By replacing the crosslinker BAP with other structures, such as MBAA, only minor differences in swelling were observed (Figure 1c), whereas the structure of the monomer had a major influence (Figure 1d). It can be noted that charged monomers in particular cause an increase in the degree of swelling, in contrast to more neutral monomers.

**Figure 2:**
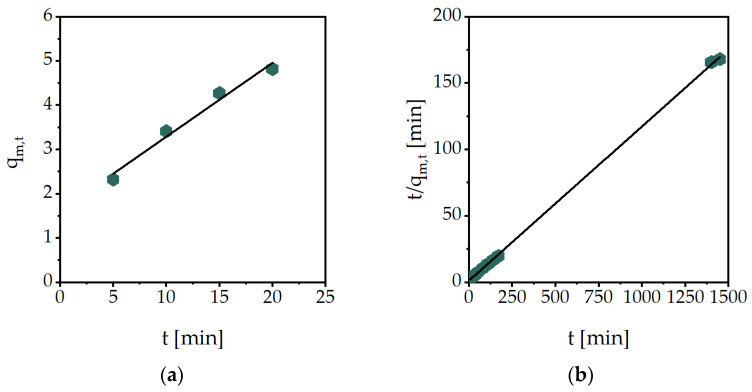
Diagrams used for further calculations exemplary for the swelling experiment of MASO_3_ (2 mol/L) and MBAA (2 mol%) as a crosslinker. Linear fits to obtain (**a**) the initial swelling rate and (**b**) the swelling rate constant *k_S_*.

From these studies, the initial swelling rate could be calculated (Figure 2a). The swelling rate constant *k_S_* was obtained from further calculations from the slope shown in Figure 2b. Additionally, the equilibrium water content (*EWC*) was calculated, representing water that was absorbed from the hydrogels in the equilibrium state. These are summarized in Table 1. All other values obtained are summarized in Table 2.

### 2.2. Mechanical Characterization

**Figure 3:**
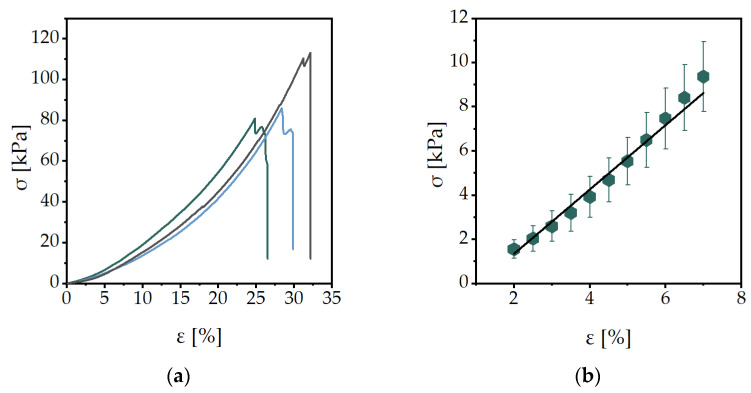
Mechanical characterization of MASO_3_ (2 mol/L) with BAP (2 mol%) as a crosslinker: (**a**) technical stress-strain curves of three single measurements with the swollen hydrogel until breakage and (**b**) linear interpolation of the data from *ɛ* = 2−7% to determine the compressive modulus.

The compressive modulus of the hydrogels, swollen in PBS to their equilibrium state, was determined from the technical stress-strain curves exemplarily shown in Figure 3a. Up to a maximum of 15% technical strain, a linear trend can be seen in this sample, which is referred to as the linear elastic range. With increasing strain, the slope becomes exponential up to a sudden drop, which marks the breakage of the hydrogel. This graph can be primarily explained by the charged side chains of the hydrogel. The greater the strain of the polymer, the closer the equally charged functional groups are to each other. This repulsion leads to increased stress with increasing strain. In addition, it can be noted that these measured samples have a slightly different maximum strain, but the curves in the linear elastic range are similar and reproducible. The compressive modulus can be calculated in the linear elastic range, using linear interpolation (Figure 3b).

During mechanical testing, several challenges emerged. For mechanical investigations, the top and bottom surfaces of the samples need to be planar for reliable results. The synthesis of the hydrogels was carried out in cylindrical molds. During free radical polymerization, the polymer samples contract to some degree, resulting in a slightly planoconcave top surface, resulting in a spreading of the sample top surface, when a small strain is put on. The specimen only exerts a smaller counterforce, which means that less stress is measured and the calculated modulus is too low. The AAMPSO_3_ hydrogel was the least impacted by these effects, giving a clear trend in the values of the compressive moduli (Table 3). It can be noted that the moduli increase with a higher monomer concentration or crosslinker amount, indicating a stiffer hydrogel. For example, with a hydrogel composition of 2 mol/L monomer concentration and 2 mol% crosslinker, the modulus increases from 537.2 ± 27.1 kPa to 841.3 ± 78.8 kPa when the crosslinker content is raised to 3 mol%. Due to its structure, the hydrogel VBTMA has extremely slow gelation, due to relatively more stable radicals [21]. As a consequence, only a smaller percentage of monomers are converted. The synthesized hydrogels are therefore extremely soft, which is reflected in a low modulus. VBTMA with a monomer concentration of 2 mol/L, for example, with BAP (2 mol%) as a crosslinker, has a modulus of 2.5 ± 0.1 kPa, which is much lower than the 537.2 ± 27.1 kPa of AAMPSO_3_. However, even in this case, an increase in the compressive modulus can be observed by higher monomer or crosslinker content.

On the contrary, MASO_3_ hydrogels measurements revealed even more challenges. These hydrogels were extremely brittle in the swollen state and breakage occurred more often while preparing the samples. Additionally, the hydrogels were affected by the volume shrinkage while polymerization, as mentioned above. As a result, these findings are still reliable at low concentrations, but not at higher concentrations.

**Figure 4:**
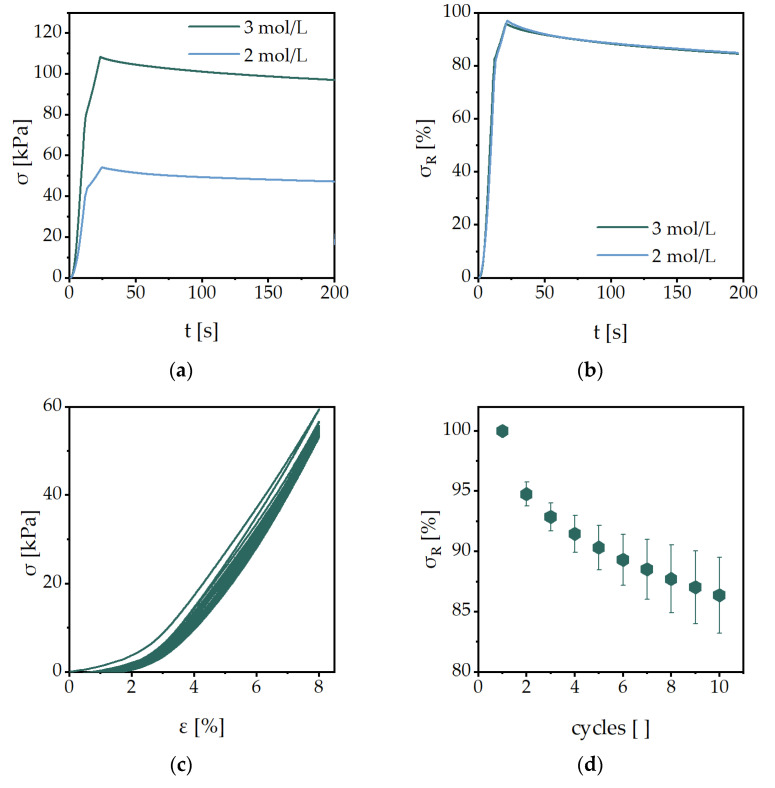
Averaged stress-relaxation profiles of the hydrogels with the monomer AAMPSO_3_ (2 mol/L) with BAP (2 mol%) as a crosslinker (*ε* = 8%; *n* = 3) (**a**) to the total stress and (**b**) the residual stress, as well as (**c**) the cyclic compression loading of one AAMPSO_3_ sample and (**d**) the normalized residual stress of the averaged AAMPSO_3_ samples.

The results of the stress-relaxation tests of the hydrogel AAMPSO_3_ with different monomer concentrations are shown in Figure 4a. Immediately after putting the sample to the technical strain of 8%, a sudden decrease was followed by a slighter decrease in stress. Ideally, the stress asymptotically approaches a certain value and then remains constant. However, these effects have not yet been observed. In the case of our samples, however, the stress continued to decrease steadily even after more than 200 s. However, this can rather be attributed to the drying effects of the hydrogel during the measurement and not to further relaxation. The drying of the hydrogel results in a slight shrinkage, causing the hydrogel to resist slightly less force. Therefore, all measurements listed here were completed after 200 s. Although the monomer concentration has a large influence on the opposing force of the hydrogel (Figure 4a), the normalized curves on the maximum force are very similar (Figure 4b). The data for the other hydrogel compositions are summarized in Table 4.

The cyclic compression loading experiments of AAMPSO_3_ hydrogels crosslinked with BAP are shown in Figure 4c. Three samples were run through ten cycles each. It is noticeable that all three experiments ran reproducibly and with only minimal deviations. In addition, it can be said that the compressive stress in the hydrogels decreases slightly per cycle, but asymptotically approaches a final value (Figure 4d). The slight decrease in the last cycles can also possibly be attributed to drying effects, as in the stress-relaxation experiments. The residual stresses of selected cycles are shown in Table 4. It can be stated that after ten load cycles, 86 ± 3% to 97 ± 6% of the residual stress of the investigated hydrogels can be examined.

### 2.3. Comparison of a Biomaterial Used for Treatment of Cartilage Lesions

**Figure 5:**
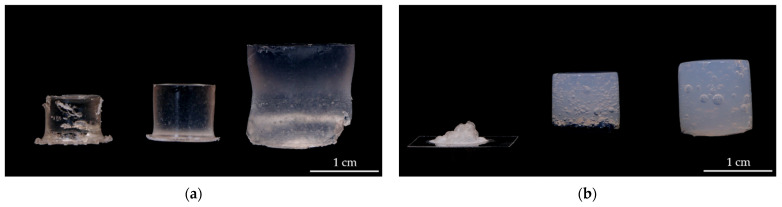
Comparison of the (**a**) synthesized hydrogels (MASO_3_ 2 mol/L, BAP 2 mol%) and the (**b**) ChondroFiller^®^ liquid biomaterial in the dried, freshly synthesized, and swollen state.

ChondroFiller^®^ liquid is a cell-free 2-component collagen type-I gel, isolated from rat tail tendons. The ChondroFiller^®^ liquid is described as a pressure-resistant gel to fill cartilage defects [22]. Therefore, this material was chosen as a reference material for our developed hydrogels based on polyelectrolytes. The main differences are found in the dried state (Figure 5). While the MASO_3_ hydrogel shrinks only slightly but retains its three-dimensional cylindrical shape, the ChondroFiller^®^ liquid collapses and undergoes severe shrinkage. When swelling, both materials retain their cylindrical shape, but the synthetic material swells more (7.7 ± 0.3) than the ChondroFiller^®^ liquid (5.3 ± 0.1). Furthermore, the synthetic materials are clear, whereas the ChondroFiller^®^ liquid is an opaque material.

**Figure 6:**
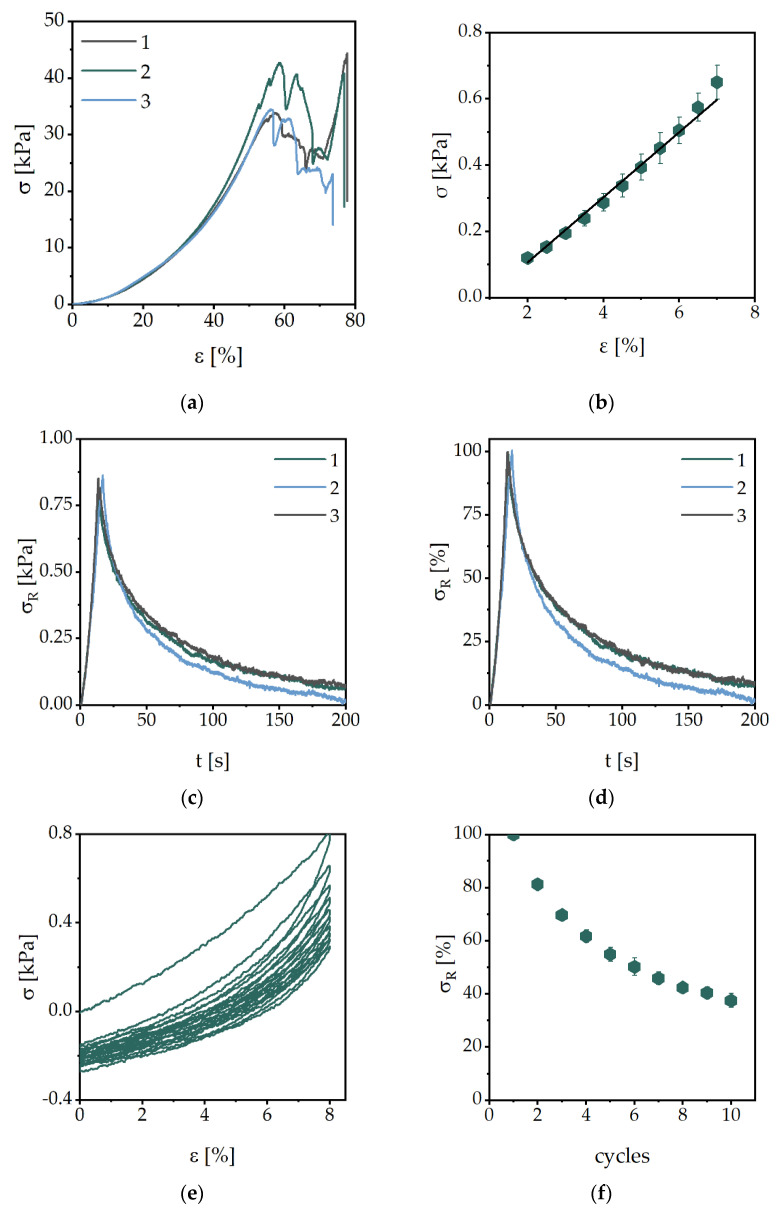
Characterization of ChondroFiller^®^ liquid with (**a**) the stress-strain curves, (**b**) the linear interpolation of the data from *ɛ* = 2−7% to determine the compressive modulus, (**c**) the stress-relaxation profile to the total stress, (**d**) the stress-relaxation experiment to the residual stress, (**e**) on of the cyclic compression loading experiments and (**f**) the normalized residual stress of each cyclic loading experiment.

The results of the mechanical characterization of the ChondroFiller^®^ liquid are shown in Figure 6. The stress-strain curves of the material were reproducible in the first 40% of the deformation (Figure 6a). Only after the breakage of the material, around 55% of the deformation, the curves differ. The resulting compressive modulus is 0.098 ± 0.0004 kPa (Figure 6b). Compared to our synthetic hydrogels, showing moduli between 2.5 ± 0.1 kPa and 1708.7 ± 67.7 kPa, the ChondroFiller^®^ liquid had significantly lower values. The results of the stress-relaxation experiments are shown in Figure 6c,d. However, the residual stress of this biomaterial is much lower compared to synthetic hydrogels. The residual stress after 200 s is 5% and 0.04 kPa, whereas the synthetic hydrogels have residual stress of around 85%. The cyclic loading experiments showed the mechanical behavior of the biomaterials comparable to the static tests (Figure 6e,f). After ten load cycles, the residual stress was around 40% and 0.3 kPa.

## 3. Conclusions

In the presented study, we successfully synthesized hydrogels, characterized their complex mechanical behavior and compared them to commercially available ChondroFiller^®^ liquid. The volumetric swelling degrees *q_v,∞_* for our hydrogels, however, are usually higher than the swelling degrees based on mass *q_m,∞_*. The swelling degree of ChondroFiller^®^ liquid is in the range of the synthetic hydrogels, whereas the ChondroFiller^®^ liquid is rather in the upper range of the synthetic swelling degrees.

We found that the synthesized polyelectrolyte hydrogels can withstand higher mechanical strength than the ChondroFiller^®^ liquid. Compared to data from human cartilage with a compressive modulus of 800 ± 333 kPa, 570 ± 170 kPa, and 310 ± 180 kPa for humeral, patellar, and femoral cartilage, respectively [23], the synthetic hydrogels showed closer values. It is also known that histopathological cartilage damage decreases Young’s modulus, because in hypertrophic cartilage a change in swelling degree often occurs [24,25]. During stress-relaxation experiments of human cartilage, almost 70% of the initially applied stress has relaxed after 150 s [22], which is more comparable to ChondroFiller^®^ liquid (80%).

In the literature, several hydrogels were described and mechanically characterized that possess a synthetic and biological polymer component. In most cases, the widely known polyacrylamide is used as the synthetic polymer, although the biological polymer part is often varied [26,27,28,29]. Pourjavadi et al. described polyacrylamide/alginate hydrogels with tunable mechanical properties, with elastic moduli up to 85.9 kPa [27]. Kanca et al. showed related polyacrylamide/alginate hybrid hydrogels with compressive moduli between 10.0 and 38.1 kPa and swelling degrees of 9.5 to 13 in PBS (pH 7.4) at 37 °C. Compared to our polyelectrolyte hydrogels, these hybrid materials exhibited a higher swelling degree and a much lower compressive modulus. However, they show similar resistance in the stress-relaxation experiments with residual stresses of 89.6 to 97.5% [29].

Hence, one possibility could be the fusion of the two materials to produce an optimal result. The synthetic hydrogel could be used as a mechanically stable core overlaid with ChondroFiller^®^ liquid. Moreover, not only do the mechanical properties have to be considered, but also properties such as biocompatibility. For this purpose, future studies should investigate cell viability and proliferation after treatment with eluted components of the hydrogels as well as the adherence ability of cells on the material surface.

This study helps with the understanding of the mechanical properties of synthetic hydrogels based on polyelectrolytes for cartilage regeneration. The results could have an important impact on mechanically driven material design in cartilage tissue engineering. Furthermore, the results in swelling behavior and mechanical characterization clearly show that these properties can be controlled and tuned according to the needs of materials as cartilage substitutes.

## 4. Materials and Methods

### 4.1. Chemicals

**Scheme 1:**
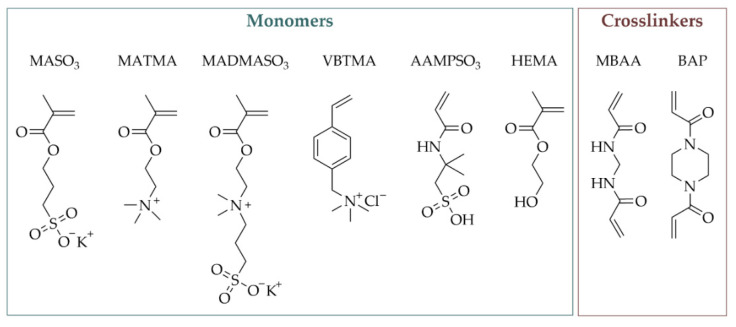
Overview of the monomers and crosslinkers used in this study.

3-Sulfopropylmethacrylate potassium (MASO_3_) (98%; Merck KGaA, Darmstadt, Germany), [2-(Methacryloyloxy)ethyl]trimethylammonium chloride (MATMA) (75 wt% in H_2_O; Merck KGaA, Darmstadt, Germany), [2-(Methacryloyloxy)ethyl]dimethyl-(3-sulfopropyl)ammo-niumhydroxide (MADMASO_3_) (95%; Merck KGaA, Darmstadt, Germany), (Vinylbenzyl)trimethylammonium chloride (VBTMA) (99%; ACROS Organics, Schwerte, Germany), 2-Acrylamido-2-methyl-1-propanesulfonic acid (AAMPSO_3_) (99%; Merck KGaA, Darmstadt, Germany), 2-Hydroxyethylmethacrylate (HEMA) (97%; Alfa Aesar, Landau, Germany), N,N’-Methylenebis(acrylamide) (MBAA) (99%; Merck KGaA, Darmstadt, Germany), N,N′-Bis-(acryloyl)-piperazin (BAP) (99%; Merck KGaA, Darmstadt, Germany), N,N, N’,N’-Tetramethylethylendiamine (TMEDA) (≥99.5%; Merck KGaA, Darmstadt, Germany) and Ammonium persulfate (APS) (98%; Roth, Karlsruhe, Germany) were used as received (Scheme 1). Additionally, ultrapure water was used throughout the study.

### 4.2. General Procedure for the Hydrogel Synthesis

**Scheme 2:**
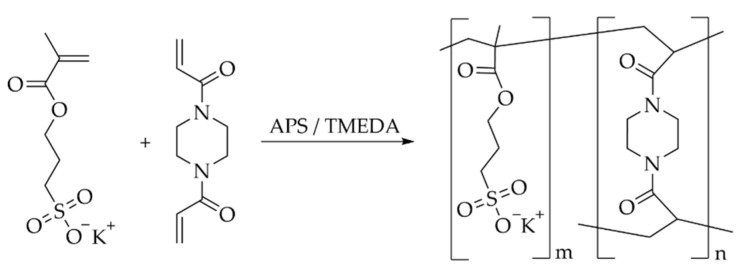
Synthesis of the polyelectrolyte hydrogels via radical polymerization, exemplary with MASO_3_ as the monomer and BAP as the crosslinker.

The hydrogels were synthesized by radical polymerization as previously described by our group in several publications [21,30,31] (Scheme 2). In short, the monomer and the corresponding amount of the crosslinker MBAA or BAP were dissolved in ultrapure water, to adjust the total monomer concentration to the required value. Afterward, the APS solution was added (0.1 mol%) and the reaction mixture was degassed for 15 min. The required amount of TMEDA was added (1.9 mol% of the total monomer concentration) to the reaction mixture and filled into cylindrical molds (10 mm height × 6 mm diameter) and stored for 24 h at h at 22 ± 2 °C.

### 4.3. ChondroFiller^®^ Liquid

ChondroFiller^®^ liquid is a two-chamber syringe system and was provided by the Meidrix Biomedicals GmbH (Esslingen, Germany). While one chamber contains collagen type-I, the other chamber is filled with a neutralization solution consisting of water, and HEPES buffer (2-[4-(2-hydroxyethyl)piperazin-1-yl]ethanesulfonic acid), NaCl, and NaOH. The material was filled in cylindrical-shaped molds to obtain hydrogels with a diameter of 6 mm and a height of 10 mm.

### 4.4. Gravimetric and Volumetric Swelling Experiments

The solvent uptake kinetics of the hydrogels were measured gravimetrically and volumetrically in PBS (phosphate-buffered saline, pH 7.4) at 36 ± 1 °C as a function of time. After drying the hydrogel samples for 48 h at room temperature on air, they were placed in a strainer and dipped in PBS. The weights of the swollen hydrogels were determined at different time points. The measurement was finished when the equilibrium state was reached. All experiments were performed as triplicates. The swelling degree *q_m,t_* was calculated using the following equation:

(1) $$q_{m,t}=\frac{M_{t}-M_{0}}{M_{0}}$$

with *M_t_* being the mass of the hydrogel at time *t* and *M*_0_ being the initial dry mass of the hydrogel at *t* = 0. For the equilibrium gravimetric swelling degree *q_m,∞_* the equilibrium mass of the hydrogel *M_∞_* was used instead of *M_t_*.

The volumetric swelling degree *q_v,t_* was determined out of the measured diameter and height of the hydrogel, using the following relation:

(2) $$q_{v,t}=\frac{V_{t}-V_{0}}{V_{0}}$$

with *V_t_* being the volume of the hydrogel at the time *t* and *V*_0_ being the initial dry volume of the hydrogel at *t* = 0. For the equilibrium volumetric swelling degree *q_v,∞_* the equilibrium mass of the hydrogel *V_∞_* was used instead of *V_t_*.

The absorbed water amount, the equilibrium water content (*EWC*) was calculated with the following term:

(3) $$EWC=\frac{M_{\infty }-M_{0}}{M_{\infty }}$$

The kinetics of the polymer swelling was investigated in more detail an according to the calculations of one of our previous studies [20]. In short, the process can be described as a second order relation:

(4) $$\frac{t}{q_{m,t}}=\frac{1}{k_{S}\times q_{m,\infty }^{2}}+\frac{1}{q_{m,\infty }}\times t$$

From the obtained slope and the y-intercepts of the linear regression of the experimental data, theoretical values of the initial swelling rates and the swelling rate constants *k_S_* could be calculated.

### 4.5. Mechanical Characterization

The characterization of mechanical properties was performed with a uniaxial testing machine (Z1.0, ZwickRoell, GmbH & Co. KG, Ulm, Germany) and a 200 N load cell (Xforce P, ZwickRoell GmbH & Co. KG, Ulm, Germany) at 20 ± 2 °C. A preload of 0.01 N was applied to the hydrogel and ChondroFiller^®^ liquid samples at the test speed of 0.05 mm/s by a previous study [32]. All measurements were performed as triplicates and analyzed with the software testXpert II (ZwickRoell GmbH & Co. KG, Ulm, Germany).

The compression measurements were carried out until the breakage of the hydrogel samples was reached. The compressive modulus *E* was able to be calculated within the linear range of the slope (*ε* = 2–7%) of the obtained technical stress-strain-curves:

(5) $$E=\frac{\sigma }{\varepsilon }=\frac{F\times l_{0}}{A\times ∆l}$$

With *A* being the idealized cross-section and *l*_0_ length of the samples in an uncompressed state, ∆*l* the technical change in length and *F* the nominal force.

For the stress-relaxation experiments, the hydrogel samples were compressed until a technical strain *ε* of 8% was reached. Afterward, the resulting position was kept constant and the nominal force was recorded for 200 s. The residual stress *σ_R_* was obtained as the fraction of stress at the end of the measurement (or a cycle) to the stress at 0 s (*ε* = 8%).

Additionally, cyclic loading experiments were performed. All samples were compressed until a technical strain *ε* of 8% was reached and relaxed to the initial position with the speed of 0.05 mm/s. The cycle was carried out 10 times.

## Figures and Tables

**Table 1:** Summary of the calculated swelling kinetic values of different hydrogels.

Monomer	Crosslinker	*c_M_* (mol/L)	*c_CL_* (mol%)	*q_m,∞_*	Initial Swelling Rate (min^−1^)	*k_S_* (10^−2^)	*EWC*
MASO_3_	BAP	2	2	7.7 ± 0.3	0.167	1.23	0.89 ± 0.01
		2.5		4.3 ± 0.1	0.082	2.48	0.81 ± 0.01
		3		4.9 ± 0.2	0.150	3.43	0.83 ± 0.01
	MBAA	2	2	7.7 ± 0.3	0.177	1.05	0.89 ± 0.01
		2.5		4.4 ± 1.3	0.113	3.17	0.81 ± 0.05
		3		4.0 ± 0.2	0.090	4.24	0.80 ± 0.01
MATMA	BAP	3	2	4.9 ± 0.1	0.102	3.38	0.45 ± 0.02
	MBAA	3	2	5.4 ± 0.4	0.065	1.24	0.84 ± 0.01
HEMA	BAP	3	2	1.0 ± 0.1	*	1.28	0.50 ± 0.03
	MBAA	3	2	0.9 ± 0.1	*	1.51	0.51 ± 0.03

* not determined.

**Table 2:** Comparison of the volumetric (*q_v,∞_*) and gravimetric (*q_m,∞_*) swelling degree.

Monomer	Crosslinker	*c_M_* (mol/L)	*c_CL_* (mol%)	*q_m,∞_*	*q_v,∞_*
MASO_3_	BAP	2	2	7.7 ± 0.3	10.7 ± 0.8
			2.5	5.0 ± 0.9	4.1 ± 0.2
			3	5.5 ± 0.2	6.8 ± 0.4
		2.5	2	4.3 ± 0.1	*
			2.5	5.1 ± 0.4	6.1 ± 0.9
			3	*	*
		3	2	4.9 ± 0.2	*
			2.5	4.6 ± 0.3	5.7 ± 0.4
			3	4.7 ± 0.9	5.2 ± 0.9
	MBAA	2	2	7.7 ± 0.3	10.7 ± 0.8
		2.5		4.4 ± 1.3	*
		3		4.0 ± 0.2	*
MATMA	BAP	3	2	4.9 ± 0.1	5.7 ± 0.2
	MBAA	3	2	5.4 ± 0.4	6.2 ± 0.2
HEMA	BAP	3	2	1.0 ± 0.1	0.6 ± 0.1
			3	0.9 ± 0.1	0.9 ± 0.1
		4	3	0.1 ± 0.0	0.1 ± 0.0
			4	0.1 ± 0.0	*
	MBAA	3	2	1.0 ± 0.1	1.0 ± 0.3
MADMASO_3_	BAP	2	2	2.8 ± 0.1	3.2 ± 0.2
AAMPSO_3_	BAP	2	2	2.1 ± 0.1	2.4 ± 0.4
			2.5	1.8 ± 0.6	1.8 ± 0.5
			3	1.7 ± 0.1	1.5 ± 0.2
		2.5	2	2.5 ± 0.1	3.0 ± 0.2
			2.5	1.8 ± 0.2	2.2 ± 0.2
			3	1.7 ± 0.1	*
		3	2	*	*
			2.5	2.2 ± 0.1	*
			3	*	*
VBTMA	BAP	2	2	7.2 ± 0.3	7.3 ± 1.5
			2.5	2.9 ± 0.4	2.3 ± 0.3
			3	4.2 ± 0.3	2.5 ± 0.2
		2.5	2	2.7 ± 0.2	2.5 ± 0.4
			2.5	4.8 ± 0.9	2.7 ± 0.1
			3	7.3 ± 0.7	5.9 ± 0.2
		3	2	5.5 ± 0.4	6.7 ± 0.4
			2.5	4.2 ± 0.3	4.3 ± 0.1
			3	5.6 ± 0.7	4.2 ± 0.1

* not determined due to unsymmetrical swelling (*q_v,∞_*) or breakage of the hydrogel while swelling.

**Table 3:** Comparison of the compressive moduli of the investigated swollen hydrogels in PBS with different monomers and BAP as a crosslinker.

*c_M_* (mol/L)	*c_CL_* (mol%)	Compressive Modulus [kPa]		
		MASO_3_	AAMPSO_3_	VBTMA
2	2	146.3 ± 9.4	537.2 ± 27.1	2.5 ± 0.1
	2.5	*	1230.2 ± 48.3	98.1 ± 13.7
	3	*	995.4 ± 83.0	129.5 ± 14.5
2.5	2	430.6 ± 20.1	569.2 ± 15.6	84.9 ± 6.0
	2.5	*	1108.6 ± 80.1	26.5 ± 1.5
	3	*	1637.4 ± 164.7	22.7 ± 1.6
3	2	353.9 ± 26.2	841.3 ± 78.8	92.3 ± 3.3
	2.5	*	1708.7 ± 67.7	107.2 ± 7.3
	3	*	*	126.5 ± 8.1

* Not determined due to brittleness and/or planoconcave top surface.

**Table 4:** Residual stress of the investigated hydrogels after different cycles of the cyclic compression loading experiments.

Monomer	*c_M_* (mol/L)	*c_CL_* (mol%)	Stress-Relaxation Residual Stress *σ_R_* [%]	Residual Stress *σ_R_* of Each Cycle (%)		
				2	5	10
AAMPSO_3_	2	2	85 ± 2	95 ± 1	90 ± 2	86 ± 3
		2.5	89 ± 1	98 ± 1	95 ± 1	93 ± 1
		3	87 ± 1	99 ± 1	97 ± 2	94 ± 2
	2.5	2	91 ± 1	98 ± 1	95 ± 2	92 ± 2
		2.5	89 ± 1	98 ± 1	95 ± 1	91 ± 2
		3	87 ± 1	97 ± 1	94 ± 2	92 ± 2
	3	2	85 ± 7	97 ± 1	93 ± 3	89 ± 3
		2.5	81 ± 6	99 ± 1	94 ± 3	90 ± 3
MASO3	3	2	88 ± 5	98 ± 2	96 ± 3	94 ± 4
VBTMA	3	2	90 ± 1	99 ± 1	98 ± 2	97 ± 6

## Data Availability

The data presented in this study are available upon reasonable request from the corresponding author.
